# Musculoskeletal pain and ergonomic risks in teachers of a federal institution

**DOI:** 10.47626/1679-4435-2020-608

**Published:** 2021-02-11

**Authors:** Kristine Kraemer, Maria Fernanda Moreira, Bruno Guimarães

**Affiliations:** Instituto Federal Catarinense Campus São Bento do Sul, Curso Técnico Integrado ao Ensino Médio em Segurança do Trabalho - São Bento do Sul (SC), Brazil

**Keywords:** teacher, ergonomics, musculoskeletal pain, occupational health

## Abstract

**Introduction:**

Ergonomic risks are a major health hazard for teachers, causing musculoskeletal pain and decreasing both their quality of life and the quality of the education offered to students.

**Objectives:**

To evaluate musculoskeletal pain and ergonomic risk factors in the workplace of teachers at the São Bento do Sul Campus of the Instituto Federal Catarinense.

**Methods:**

Twenty-five teachers completed sociodemographic and ergonomic risk questionnaires, as well as the Nordic Musculoskeletal Questionnaire. The furniture and equipment at their workstations were also evaluated using a checklist.

**Results:**

Seventy two percent of teachers were male, and the mean age of the sample was 37.08±7.14 years. In response to the questionnaires, 72% of participants reported little knowledge of ergonomics and 68% said they did not apply these principles in their daily life. The main ergonomic risks to which teachers were exposed were prolonged sitting and standing, sharp corners on desks, use of laptop touchpads and inadequate monitor height. All teachers reported pain in the past 12 months, with the most frequently affected areas being the low back (60%), neck (56%) and shoulders (48%).

**Conclusions:**

These findings highlight the importance of ergonomic adaptations and changes in the work habits of teachers in order to improve their health and quality of life, while also allowing them to deliver higher-quality education to their students.

## INTRODUCTION

Work is a major part of life. However, when performed incorrectly, it can also be harmful to health. The occupational characteristics of some workers may render them especially susceptible to work-related musculoskeletal pain.^1^ These issues affect workers across several occupations and are among the main causes of work-related disability.^2^

Risk factors for musculoskeletal disorders include occupational factors such as mechanical compression, excessive force, repetitive movements, long hours and inadequate posture, as well as individual characteristics such as smoking, obesity, organizational features and psychosocial issues.^3^ Some of these risk factors may be hazardous to the health of teachers, whose work involves repetitive movements such as writing, in addition to prolonged standing, repetitive tasks like grading, as well as daily computer use.^4,5^ Teachers often use computers to perform administrative tasks, prepare classes, develop teaching strategies, write up research and extension projects, and develop curriculum plans. The main causes of illness and disability among teachers include mental and behavioral conditions, respiratory diseases and musculoskeletal disorders.^^5^,6^

Several organizations, including educational institutions, fail to meet the ergonomic principles outlined in regulation number 17 (Ergonomics), which establishes a series of guidelines to ensure adequate working conditions.^7^ This issue becomes particularly relevant given the relationship between inadequate working conditions, musculoskeletal symptoms, poor psychosocial well-being, and low quality of life among teachers.^8^

The combined assessment of musculoskeletal symptoms and occupational activities associated with ergonomic risk can give rise to causal hypotheses, which can guide studies and strategies to improve current working conditions.^9^ Initiatives aimed at supporting teachers and providing a context-sensitive understanding of their problems can also be implemented by schools and government sectors in order to increase teacher empowerment. There is also a need for strategies to improve the working conditions and prevent the health hazards to which teachers are exposed, thereby improving the quality of the education they offer their students.^10^

The influence of individual and environmental variables on the health of teachers has only been sparsely studied in Brazil.^11^ There is also a need for a better understanding of musculoskeletal pain in teachers, and its association with the biological, ergonomic, occupational, and psychosocial aspects of teaching.^12^

While previous studies have examined musculoskeletal pain and associated risk factors in university professors^5,13,14^ and school teachers,^1,2^ these variables have not been examined in vocational teachers. Such studies are necessary since the occupational characteristics of these individuals differ from those of teachers working at other locations, such as universities and schools. Vocational teachers in federal institutes deliver professional training, secondary-level technical education, alternate educational programs (National Program of Professional Integration; Programa Nacional de Integração da Educação Profissional [PROEJA]), as well as undergraduate and graduate courses. These teachers are also involved in research and extension activities. The aim of this study was therefore to evaluate musculoskeletal pain and ergonomic risk factors in the workplace of teachers at the São Bento do Sul Campus of the Instituto Federal Catarinense (IFC).

## METHOD

A quantitative descriptive exploratory study was conducted at the São Bento do Sul Campus of the IFC. The population consisted of the 42 teachers working at the institution. Inclusion criteria for this study were not being on leave at any point in the study period, having a 40-hour or exclusive contract with the institution and providing written consent to participate in the study. Exclusion criteria were being employed at the São Bento do Sul Campus of the IFC for less than 6 months, working 20 hours a week or not signing the informed consent form. Seventeen teachers were excluded based on the aforementioned criteria, and as such, the final sample for the present study consisted of 25 teachers. This investigation was approved by the Human Research Ethics Committee of the IFC (CAAE No. 14598219.3.0000.8049) and conducted according to current legislation.

Data were collected from August to September 2019. Participants completed a sociodemographic questionnaire and an ergonomic risk survey, both of which were developed by the researchers of the present study. The sociodemographic questionnaire was used to assess variables such as age, weight, height, time since admission to the IFC, body mass index (BMI), and physical activity levels. BMI was calculated by dividing the weight of each participant (in kg) by the square of their reported height (in cm).^15^ The ergonomic risk questionnaire investigated the knowledge and application of ergonomic principles in everyday activities, as well as the characteristics of occupational tasks, such as frequency of computer and blackboard use, and posture during these activities. The study also involved on-site observation and the use of a checklist developed by the research team to collect information on the furniture and equipment at the workstation of each participant.

The 12-month prevalence of musculoskeletal pain was assessed using the Nordic Musculoskeletal Questionnaire (NMQ), originally developed by Kuorinka et al.^16^ and later translated and validated for use in Brazil.^17^ The NMQ is a self-administered questionnaire containing a body map divided into nine anatomical regions: (I) neck; (ii) shoulders, (iii) upper back, (iv) elbows, (v) wrist/hands, (vi) low back, (vii) hips/thighs, (viii) knees and (ix) ankles/feet.^16^ The participant is asked to report the frequency with which they felt pain or discomfort in each region in the previous 12 months and the previous 7 days, as well as any functional limitations and consultations with health professionals due to musculoskeletal symptoms in the previous 12 months.^16^

Data were analyzed in Microsoft Excel^®^, 2019, using descriptive statistics.

## RESULTS

The sample consisted of 25 teachers, 18 of whom (72%) were male while 7 (28%) were female. Ages ranged from 28 to 56 years, with a mean of 37.08 (SD, 7.14) years. Marital status was reported as single by 36% of participants, divorced by 4% and married by 60% of participants. Forty percent of participants had children. While 48% of participants had master’s degrees, 20% had specialist degrees, 20% had doctorates and 12% had completed postdoctoral training. Questions regarding participants’ knowledge of ergonomics revealed that 8% of the sample reported no knowledge of the topic, while 72% reported some knowledge, 16% had a reasonable amount of knowledge and 4% reported extensive knowledge of ergonomic principles. Yet only 32% of participants reported applying these principles in their daily practice, which was not the case for the remaining 68% of the sample. The data collected using the sociodemographic questionnaire are shown in Table 1.

**Table 1:** Sociodemographic characteristics of the sample.

	Mean	Standard deviation
Height (m)	1.76	0.11
Weight (kg)	82.12	17.65
BMI	26.52	5.59
Teaching experience (years)	10.08	6.82
Number of classes taught	6.20	2.12
Weekly class hours in the second half of 2019	15.59	6.11
	**Yes**	**No**
Current physical activity	64%	36%

BMI: body mass index.

Data from the ergonomic risk questionnaire are shown in Table 2, while the results of the checklist are presented in Table 3. According to these instruments, the most common ergonomic risk factors in the sample were prolonged sitting and standing, sharp corner on work desks, use of a laptop touchpad instead of a mouse and improper screen height.

**Table 2:** Ergonomic risk questionnaire.

	0-19%	20-39%	40-59%	60-79%	80-100%
Percent of class time spent using overhead projector	36	20	16	8	20
Percent of class time spent standing	0	0	8	12	80
Percent of work time outside the classroom spent on computer	0	12	4	36	48
Percent of class time spent writing on blackboard	20	36	16	12	16
		**Top-middle**	**Middle**	**Middle-bottom**	**Bottom**
Part of the blackboard usually used	-	28	40	28	4

Data presented as %.

**Table 3:** Checklist results.

	Yes	No
Feet touch the floor	83.33	16.66
Chair height adequate	62.50	37.50
Chair height adjustable	100	0
Backrest height adequate	62.50	37.50
Sharp corners on desk	91.66	9.16
Desk at elbow height	54.16	45.83
Desk has room for forearm support	100	0
Use of laptop touchpad	58.33	41.66
Mouse pad with wrist support	4.16	95.83
Use of laptop keyboard	83.33	16.66
Keyboard with wrist support	29.16	70.83
Upper edge of computer monitor at eye level	12.50	87.50

Data presented as %.

The NMQ revealed that 100% of teachers reported feeling pain at some point in the past 12 months. The most frequently affected areas were the low back (60%), neck (56%), shoulders (48%), upper back (40%), wrists/hands (32%), hips/thighs (28%), knees (24%), ankles/feet (20%) and elbows (8%).

Further analyses were then conducted in order to investigate whether any sociodemographic characteristics, ergonomic risk factors or items in the ergonomic checklist were associated with the occurrence of pain in each of the regions assessed by the NMQ. The results of these procedures are shown in Table 4.

**Table 4:** Association between pain, sociodemographic variables, ergonomic risks, and the observational checklist.

	Low back	Neck	Shoulders
Age			
25-35	60	42.86	50
36-45	20	35.71	33.33
46-56	20	21.43	16.66
Height (m)			
1.55-1.75	46.67	71.43	66.66
1.76-1.85	40	28.57	25
1.86-1.98	13.33	0	8.33
BMI			
18-24 (normal weight)	40	42.86	66.66
25-29 (overweight)	60	35.71	16.66
Over 30 (obesity)	26.67	21.43	16.66
Teaching experience (years)			
3-10	53.33	64.29	58.33
11-20	40	28.57	33.33
20-30	20	7.14	8.33
Number of classes taught			
3-6	66.67	50	41.66
7-9	33.33	50	58.33
Weekly class hours in current semester			
10-15	60	71.43	75
16-20	20	21.43	16.66
Over 21	20	7.14	8.33
Current physical activity	60	78.57	90
Knowledge of ergonomic principles			
None	13.33	14.28	0
Some	66.66	78.57	83.33
Reasonable	20	7.14	16.66
Does not apply ergonomic principles to daily practice	66.66	78.57	66.66
Percentage of class time spent writing on blackboard			
0-19	13.33	28.57	41.66
20-39	26.67	28.57	25
40-59	26.67	21.43	16.66
60-79	13.33	14.29	8.33
80-100	20	7.14	8.33
Part of the blackboard usually used			
Top-middle	73.34	57.14	66.66
Middle	20	42.86	33.33
Middle-bottom	0	0	0
Bottom	6.67	0	0
Percent of class time spent standing			
60-79	13.33	21.43	16.66
80-100	86.67	78.57	83.33
Percent of work time outside the classroom spent on computer			
20-39	6.67	7.14	0
40-59	6.67	0	0
60-79	26.67	42.86	66.66
80-100	60	50	33.33
Chair height not adequate	33.33	57.14	50
Backrest height not adequate	40	42.86	41.66
Desk not at elbow height	33.33	42.86	50
Use of laptop touchpad	80	57.14	50
Use of laptop keyboard	93.33	92.86	91.66
Upper edge of computer screen not at eye level	93.33	100	100

BMI: body mass index. Data presented as %.

## DISCUSSION

In the present study, 72% of teachers were male. This differs from previous studies such as those of Gomes et al.,^18^ involving university professors, and Oliveira et al.,^19^ who looked at professors in federal institutes, where 56.14 and 55.80% of teachers were female, respectively. This may be because the specific courses offered at the São Bento do Sul IFC Campus (professional training in Occupational Safety, Information Technology and Industrial Automation, Computer Engineering, Industrial Automation and Control Engineering) traditionally attract a higher proportion of male students and teachers.

Participants ranged in age from 8 to 56 years, with a mean of 37.08 (SD, 7.14) years. These results are similar to those obtained by Oliveira et al.,^19^ who found that 67.45% of a sample of teachers at a federal institute were younger than 40 years. Similarly, the university professors studied by Sanchez et al.^5^ had a mean age of 34.89 (SD, 7.23) years. These findings differ, however, from those of Vedovato and Montreiro^4^ whose sample of state school teachers in São Paulo had a mean age of 41.4 (SD, 9.4) years.

Participants in the present sample had a mean of 10.08 (SD, 6.82) years of teaching experience. This is similar to previous studies. Sanchez et al.^5^ investigated university professors with a mean of 9.75 (SD, 8.69) years of experience, and Ceballos and Santos^12^ assessed a sample of kindergarten and primary teachers where most participants (58.30%) had less than 10 years of experience in the field. Similarly, 53.49% of the federal institute teachers studied by Oliveira et al.^19^ had been teaching for 5 to 15 years.

The mean teaching time per week was of 15.59 (SD, 6.11) hours, corroborating previous findings in the literature. Oliveira et al.,^19^ for instance, found that 60.47% of teachers in their sample taught for 12 to 24 hours every week. Machado and Limongi,^10^ however, found that municipal school teachers in the city of Uberlândia, Minas Gerais, spent a mean of 30 hours in the classroom every week. This may be because the workload of IFC teachers includes the time spent preparing classes, grading activities and exams, as well as up to 10 hours a week for research and extension activities.

The main ergonomic risk factors identified in this study were prolonged standing and sitting, sharp edges on work desks, use of a laptop touchpad instead of a mouse and inadequate screen height (upper edge of the monitor below eye level). These results are in line with previous studies, which found that teachers are exposed to a variety of ergonomic risk factors, including repetitive movements, frequent computer use, prolonged standing^4^ and siting,^20^ significant physical effort and repetitive activities.^1^ Poor ergonomic working conditions have been found to be associated with health issues in teachers.^1,6,7^

All participants in the present sample experienced pain at some point in the previous 12 months, much like the university professors surveyed by Sanchez et al.^5^ and Mota et al.^8^ The regions most frequently affected in the present study were the low back (60%), neck (56%) and shoulders (48%). These findings agree with those of Sanchez et al.,^5^ who found that the incidence of pain in university professors was highest in the low back (80.56%), neck (77.78%) and shoulders (72.22%). Similarly, in a study by Lima Júnior and Da Silva,^14^ federal university professors were most likely to report pain in the low back (54.8%), upper back (45.20%) and shoulders (23.80%). Suda et al.^13^ also surveyed university professors and found that 70% reported neck pain while 64% experienced low back pain in the previous 12 months. In a study conducted by Mota et al.^8^ at the Universidade Estadual do Sudoeste da Bahia, the prevalence of neck and low back pain was 44.80%, while that of shoulder pain was 36.70%.

The analysis of sociodemographic variables and NMQ data revealed that 60% of the teachers who reported low back pain had a BMI greater than 25 and would therefore be classified as overweight or obese according to the World Health Organization (WHO).^21^ These findings agree with previous studies suggesting an association between low back pain, high BMI, overweight and obesity.^3^,^22^ It is possible that the excess weight may place additional strain on the osteoarticular system, altering the biomechanical balance of the body and increasing the risk of chronic low back pain in individuals classified as overweight or obese.^15^

Additionally, 80% of teachers who reported low back pain taught 10 to 20 hours a week and used a computer for 60 to 100% of the remaining work hours. Participants in this study worked 40 hours a week, and as such, may spend up to 30 hours a week working on their computers. Prolonged sitting is a risk factor for low back pain and disability,^^23^-25^ since this position leads to prolonged lumbar flexion, reduced lumbar lordosis, and static overload of muscles in the lower back, all of which are directly related to the onset of lower back pain.^26^

Neck pain was reported by 56% of the sample. This may be attributable to improper monitor height, since 100% of participants did not keep their computer screens at eye level, thereby leading to sustained neck flexion for as long as these devices were in use. Additionally, since 71.43% of teachers spent 10 to 15 hours in the classroom every week, and 92.86% of those who reported neck pain used computers for 60 to 100% of the hours worked outside the classroom, they may spend up to 30 hours a week with the neck in a flexed position. Prolonged neck flexion leads to muscle fatigue and neck pain^27^ as a result of the increased gravitational load on cervical extensor muscles, leading to a greater likelihood of neck pain^28^ as a result of improper monitor height.^^29^^

Shoulder pain was also reported by 48% of teachers, none of whom kept their computer screens at eye level while working. Our results also showed that 75 to 100% of teachers who reported shoulder pain taught 10 to 15 hours a week and used a computer for 60 to 100% of work hours spent outside the classroom. These findings agree with those of several previous studies^30^ which have identified a consistently higher prevalence of shoulder pain in computer users.^17^,^^29^^,^30^ Some postures and work activities are actually considered to be risk factors for the development of musculoskeletal neck and shoulder disorders.^30^ A sustained forward head posture and/or constant neck flexion can cause static overload of neck and shoulder muscles,^30^ which combined with the repetitive movements associated with using a mouse, touchpad or keyboard can increase the likelihood of shoulder pain.

Another factor which may have influenced these findings is the fact that 83.33%, 78.57%, and 66.66% of the teachers who experienced pain in the shoulders, neck, and low back, respectively, claimed to have little knowledge of ergonomics. Additionally, 78.57% of teachers who reported neck pain and 66.66% of those with shoulder and low back pain did not apply ergonomic principles in their daily practice. An increased awareness of ergonomic risks may therefore constitute a promising strategy to prevent musculoskeletal pain in teachers, as it may lead them to change their habits by introducing pauses in the work day, avoiding prolonged sitting, and making ergonomic adjustments to the furniture and equipment at their work stations.

The present findings highlight the importance of identifying risk factors and making ergonomic adjustments to work stations^30^ in order to prevent work-related musculoskeletal disorders. In addition to preventing health hazards, strategies to improve teachers’ working conditions could also increase their quality of life and the quality of the education offered to students.^10^ Reductions in absenteeism, presenteeism, turnover, early retirement and medical leave would also reduce costs for the government and have several benefits for society as a whole.^10^ Studying the work of teachers could also provide crucial information to reduce inequality.^4^

This study has some limitations, such as our inability to conduct more sophisticated data analyses due to our small sample. Nevertheless, this is the first study to investigate ergonomic risks and the prevalence of pain in federal institutes teachers, whose occupational characteristics are known to differ from those of teachers in schools and universities. Future studies should recruit larger samples in order to investigate ergonomic risks and prevalence of musculoskeletal pain in larger groups of federal institute teachers.

## CONCLUSIONS

The present study found that federal institute teachers were exposed to a series of ergonomic risk factors, the most prevalent of which consisted of prolonged sitting and standing, sharp edges on work desks, use of a laptop touchpad instead of a mouse and improper screen height.

The 12-month prevalence of musculoskeletal pain was also significant, as all teachers reported feeling pain in at least one body within the past year. The most frequently affected body parts were the low back, neck and shoulder, which could be related to an elevated BMI, indicative of overweight and/or obesity, as well as prolonged sitting, constant neck flexion due to improper computer monitor height, as well as a lack of knowledge and application of ergonomic principles in everyday activities.

The high prevalence of pain and ergonomic risk factors identified in the present study highlight the need for changes in the work habits of teachers, as well as increased awareness of ergonomic risks. These modifications, together with adjustments to teachers’ work environments, could help decrease the prevalence of musculoskeletal pain and related consequences such as disability, reduced quality of life and low-quality education.
